# Encoding of temporal intervals in the rat hindlimb sensorimotor cortex

**DOI:** 10.3389/fnsys.2012.00067

**Published:** 2012-09-26

**Authors:** Eric B. Knudsen, Robert D. Flint, Karen A. Moxon

**Affiliations:** ^1^School of Biomedical Engineering Science and Health Systems, Drexel UniversityPhiladelphia, PA, USA; ^2^Department of Neurobiology and Anatomy, Drexel University College of MedicinePhiladelphia, PA, USA

**Keywords:** encoding, hindlimb, rat, sensorimotor cortex, temporal intervals

## Abstract

The gradual buildup of neural activity over experimentally imposed delay periods, termed climbing activity, is well documented and is a potential mechanism by which interval time is encoded by distributed cortico-thalamico-striatal networks in the brain. Additionally, when multiple delay periods are incorporated, this activity has been shown to scale its rate of climbing proportional to the delay period. However, it remains unclear whether these patterns of activity occur within areas of motor cortex dedicated to hindlimb movement. Moreover, the effects of behavioral training (e.g., motor tasks) under different reward conditions but with similar behavioral output are not well addressed. To address this, we recorded activity from the hindlimb sensorimotor cortex (HLSMC) of two groups of rats performing a skilled hindlimb press task. In one group, rats were trained only to a make a valid press within a finite window after cue presentation for reward (non-interval trained, nIT; *n* = 5), while rats in the second group were given duration-specific cues in which they had to make presses of either short or long duration to receive reward (interval trained, IT; *n* = 6). Using perievent time histogram (PETH) analyses, we show that cells recorded from both groups showed climbing activity during the task in similar proportions (35% IT and 47% nIT), however, only climbing activity from IT rats was temporally scaled to press duration. Furthermore, using single trial decoding techniques (Wiener filter), we show that press duration can be inferred using climbing activity from IT animals (*R* = 0.61) significantly better than nIT animals (*R* = 0.507, *p* < 0.01), suggesting IT animals encode press duration through temporally scaled climbing activity. Thus, if temporal intervals are behaviorally relevant then the activity of climbing neurons is temporally scaled to encode the passage of time.

## Introduction

How the brain internally represents the passage of time and subsequently uses this representation to guide behavior remains an open question (Durstewitz, 2004; Buhusi and Meck, 2005; Buonomano and Laje, 2009). The brain is able to internalize timing over a wide range of timescales through a number of different mechanisms. At one extreme of this range, circadian timing allows for the guiding of the behavior of sleep-wake cycles (Reppert and Weaver, 2002), appetite (Kalra et al., 1999), and migratory patterns (Froy et al., 2003). At the other extreme, millisecond timing is a crucial component of unconscious motor control (Ivry et al., 1988; Bijsterbosch et al., 2011), anticipation of movement consequences (Knolle et al., 2012); and speech processing in humans (Mauk and Buonomano, 2004; Schirmer, 2004; Schmitz-Hubsch et al., 2011). At more intermediate timescales, which is the focus of this paper, interval timing, ranging from seconds to minutes, is critical to conscious animal behavior such as foraging (Wallace et al., 2006; Salwiczek and Bshary, 2011), decision making (Puumala and Sirvio, 1997; Donner et al., 2009), and conscious time estimation (Rakitin et al., 1998; Mita et al., 2009). Experimental evidence suggests that distributed cortico-striato-thalamic circuits comprise the interval timing subsystem of the brain (Buhusi and Meck, 2005). Neuroimaging (fMRI) studies in human subjects highlight the recruitment of these areas during timing dependent experimental tasks (Schubotz et al., 2000; Harrington et al., 2004). Numerous animal studies have correlated neuronal modulations in cortical, striatal, and thalamic areas to behavioral timing at this time scale. Of these, the majority of studies have reported that neurons within these areas display patterns of climbing activity (Durstewitz, 2004) during task-related delays.

This climbing activity, in which neurons, on average, monotonically increase or decrease their firing rate, has been observed in premotor cortex (Crammond and Kalaska, 2000; Lebedev et al., 2008; Merchant et al., 2011), supplementary motor area (Mita et al., 2009), primary motor cortex (Roux et al., 2003; Lebedev et al., 2008), posterior parietal cortex (Janssen and Shadlen, 2005; Maimon and Assad, 2006), prefrontal cortex (Goldman-Rakic, 1995; Lebedev et al., 2004; Genovesio et al., 2009), inferotemporal cortex (Reutimann et al., 2004), striatum (Schultz et al., 1997; Chiba et al., 2008) and thalamus (Komura et al., 2001) under many different experimental paradigms. These tasks include duration discrimination (Roux et al., 2003), reaction time (Riehle and Requin, 1993; Narayana and Laubach, 2009), and delayed-response movements (Kalenscher et al., 2006; Lebedev et al., 2008; Mita et al., 2009). Thus, climbing activity has been implicated in a number of processes including motor preparation (Romo and Schultz, 1987), motor timing (Lebedev et al., 2008), working memory (Goldman-Rakic, 1995; Miller et al., 1996), decision making (Kim and Shadlen, 1999; Kalenscher and Pennartz, 2008; Sugrue et al., 2005), and anticipation of reward (Schultz et al., 1992). Importantly the rate of climbing may change in proportional to the length of experimentally imposed delay periods suggesting a mechanism for encoding the duration of time (Renoult et al., 2006; Lebedev et al., 2008). This phenomenon is generally referred to as temporal scaling (Renoult et al., 2006; Lebedev et al., 2008). Of the studies listed, however, a majority involving motor action have been primarily interested in forelimb use as the external reflection of behavioral timing. While it is likely that the hindlimb cortex also participated in temporal coding, it is currently unknown how encoding of temporal intervals occurs within the hindlimb sensorimotor cortex (HLSMC) during skilled motor output.

Similarly, it is unknown how behavioral training affects temporal encoding. Roux et al. (2003) compared the activity of neurons in MI of a monkey performing two contextually different (albeit with similar motor demands) time estimation tasks. Using these tasks, the authors were able to study neuronal firing during both the elapsed time between a preparatory signal and a temporally uncertain response signal, and in precise temporal estimation when the duration to be estimated was known at the start of the trial. It was shown that “tonic activation” (climbing activity) of neurons occurred only when the probability of performing a particular movement was 1, in other words, when the duration to be estimated was known. This neuronal behavior suggests that temporal encoding using temporal scaling occurs in a contextual manner in which an *a priori* knowledge of specific intervals in necessary. Furthermore, it implies that climbing activity is not an abstract metric of time estimation in M1, but rather a reflection of the estimation process within the context of the task itself. Merchant et al. (2011) further demonstrated the context dependence of climbing activity in neurons in the medial premotor areas (preSMA and SMA) of the monkey using synchronized- and reaction time-based finger tapping tasks. The authors demonstrated that preSMA and SMA neurons show ramping (climbing) activity that encodes the time elapsed or the time remaining between taps only when the monkeys' taps were entrained to periodic intervals; when the monkeys were reacting to an aperiodic series of auditory stimuli, no temporally-scaled timing activity was evident.

However, while each of the studies observing climbing activity are dependent upon the context of the behavioral paradigm being used (Roux et al., 2003; Merchant et al., 2011), there has not been a direct comparison between the effects of behavioral training on the encoding of temporal intervals in the cortex. To address this, in this study, we recorded activity from the HLSMC of two groups of animals performing a skilled hindlimb press task. In one group, animals were trained only to a make a valid press within a finite window after cue presentation for reward (non-interval trained, nIT), while animals in the second group were given duration-specific cues in which they had to make presses of either short or long duration to receive reward (interval trained, IT). Although nIT animals tended to make shorter duration presses, there was a sufficient number of longer duration presses to allow comparisons between groups to test the hypothesis that neurons within the HLSMC of animals trained to produce specific press durations will encode these durations through patterns of climbing and descending activity, while neurons of animals who spontaneously produce the same motor output, do not.

## Methods

### Overview of study

In this study two groups of animals were trained to press a pedal with their hindlimb in response to a GO cue. One group of animals, the nIT group, were rewarded only for making a complete press within a finite (3 s) window following GO. The second group, or IT group, were also trained to make the hindlimb press, however, animals were required to make either short (<1 s) or long (>1.5 s) duration presses in response to the specific cue delivered. Once animals were trained to proficiency, we chronically implanted arrays of microelectrodes bilaterally into the HLSMC and recorded neuronal activity while animals performed either the IT or nIT task. Offline we compared the distribution of the durations of the presses across the two groups and selected a subset of rewarded presses from each group to ensure an equivalent distribution of press durations between groups. Using these subsets of presses, we compared the single neurons response properties between the long and short presses and between the two groups. We also identified cells that displayed climbing activity in their trial averages and compared the proportion of cells displaying climbing activity across groups. To assess the role of this activity in temporal scaling, differences in the parameters of the climbing activity (e.g., slope, *R*^2^) were compared between short and long duration presses for each group separately. Finally, we used different single-trial decoding techniques to assess the role of temporal scaling in the encoding of information about press duration and the role of training.

### Skilled hindlimb reaching task

Eleven adult male Long-Evans rats (8 weeks old, 150–200 g) were trained in the skilled hindlimb press task. Animals were housed separately on a 12/12 h light/dark cycle. One week prior to training, animals were allowed restricted access to water (100 mL/kg/day) to allow for training. Access to food was *ad libitum* though animals rarely ate in the absence of water. All procedures were conducted under the approval of the Institutional Animal Care and Use Committee of Drexel University.

**Figure 1:**
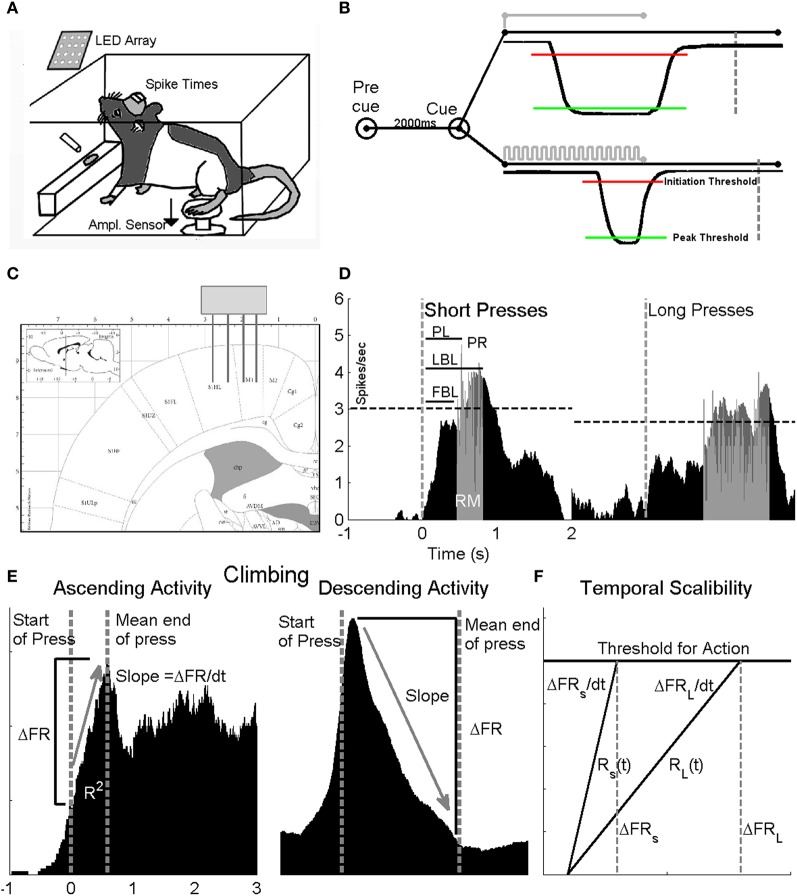
**Experimental design**. Two groups of animals were trained to press a pedal with their hindlimb in response to GO cues for a water reward. **(A)** Neural activity from the HLSMC was recorded from animals performing the task in a closed behavioral chamber and stored for off-line analysis. **(B)** The first animal group (non-interval trained) was trained with a single GO cue and the duration of the press was not relevant for reward (no image shown). The second group (interval trained) was trained to give a long duration press (>1.5) in response to a solid light cue (top trace) and a short press (<1) in response to a flashing light cue (bottom trace). Both groups of animals were required to sit quietly for a 2 s pre cue period, otherwise, a false positive was generated and the trial ended. For presses to be valid, the amplitude sensor was required to cross an initiation threshold (red line) and remain below a peak threshold (red line) for the cued duration before returning to baseline. If successful, animals were rewarded at fixed period point (IT: short: 750 ms, long: 500 ms after end of press; nIT 500 ms after end of press) following completion of correct press duration (dashed lines). Both groups of animals made short and long presses. **(C)** Stereotaxic location of 4 × 4 microelectrode array implants in rat HLSMC (from Paxinos and Watson, 2007). **(D)** To assess differences in the neuronal responses, perievent time histograms (PETHs) around the time of cue presentation (dashed vertical line) were generated for each cell for short and long duration presses separately. Neurons were considered to be modulated by the task if their firing rate exceeded the 99% confidence interval of the background firing (dashed horizontal line) rate of the smoothed response. From each PETH, measures of latency (peak—PL, first bin—FBL, and last bin—LBL), peak response (PR) and response magnitude (RM) are derived from unsmoothed histograms in the response window (gray). **(E)** To determine if cells displayed climbing activity, cells that displayed ascending (left) and descending (right) activity in their PETH were further analyzed. Measures of R2, ΔFR and slope between the start of press (left most dashed line) and the mean end of press (right most dashed line) were measured for short and long presses. **(F)** Diagram to describe temporal scaling. Temporal scaling is evident when the average change in firing rate between the short (S) and long (L) press (ΔFRS compared to ΔFRL) remains constant while the rate at which the firing rates changes (ΔFRS/dt compared to ΔFRL/dt) varies in proportion to the estimated duration.

Animals were trained in a custom-made Plexiglas chamber containing an inlet for water delivery and a moveable pedal connected to an amplitude sensor which uses a linear variable differential transformer (LVDT; Figure 1A). While learning the skilled hindlimb task, animals were rewarded for pressing the amplitude sensor via the pedal past a fixed threshold. An overhead LED array was used to provide a cue that signaled the animal to perform the proper type of press. All event times (cue onset times, reward times, amplitude data, etc.) for each session were recorded and saved via a custom LabView (National Instruments, Austin, TX) virtual instrument for offline processing.

Animals were operantly conditioned to make hindlimb level presses in response to the cue. Details of the training protocol have been published previously (Knudsen et al., 2011). Briefly, two groups of animals were trained under one of two variants of the hindlimb press task. Animals were unrestrained in the training chamber, except the tethered connection between implanted microelectrodes and the pre-amplification hardware. Over the course of behavioral training animals learn to position themselves facing toward the water inlet and away from the pedal. When a cue was delivered animals lifted one hindpaw onto the pedal, pressed and then removed the paw, placing it back onto the floor of the chamber within the required time to receive reward. In the nIT task, animals were presented with a GO cue at which point they were required to make a hindlimb press and release within a 3 s response window to receive a water reward 500 ms after release (Manohar et al., 2011). No press duration requirements were imposed on the animals, merely that they make a valid press within the response window. Performance in the nIT task was quantified by true positive rate (TPR); the total number of valid presses out of the total number of cues delivered, and the false positive rate (FPR); the total number of spurious presses outside of the trial windows.

We trained the second group of animals in the IT variation of the skilled hindlimb task. For this task, animals were trained to make either a short duration (<1 s) or long duration (>1.5 s) press when presented with flashing light or solid light cues, respectively (Figure 1B). Performance was similarly tracked with TPRs for each cue type, the overall TPR, and the false alarm rate (FAR), which was defined as the total number of spurious presses within a 2-s pre-cue period. This period insured that animals were quiescent and waiting for a set period of time before cue presentation. A single trial was defined from the time of cue onset to either reward delivery for a valid trial or to the time of lights out for an invalid trial. Press onset was defined as movement of the pedal across an initiation threshold, defined as mean of the baseline amplitude sensor reading (in volts) minus three times the standard deviation of the baseline noise floor (Figure 1B). The end of press was defined as the return of the pedal across this same threshold toward baseline. The duration of press was the time between start and end of press. To receive reward, each press was required to meet an amplitude requirement, such that the press crossed a peak threshold which was set at the mean maximum press amplitude (in volts) plus three times the standard deviation of the maximum press. For a long press cue, the trial was considered valid if and only if the amplitude of press crossed the peak threshold and remained below this threshold for the duration of the press. Any deviation of press that re-crossed the peak threshold before the end of press was considered an invalid trial, which triggered a lights out condition where the animal received no reward and was required to wait until the start of a new trial. For a short cue, the press was only considered valid if the amplitude sensor crossed the peak threshold and returned back to the initiation threshold within 1 s. If the amplitude of press did not return within 1 s, the trial was considered invalid and the lights out condition was triggered. Both groups of animals were trained until proficient in the task (nIT: >90% TPR, <10% FPR; IT: >80% TPR for both press types, <20% FAR), at which point they were ready for microelectrode implantation. Table 1 summarizes each phase of behavioral training.

**Table 1:** **Training schedule for skilled hindlimb press task**.

**Phase**		**Level**	**Training criterion**	**Mean number of sessions**
I	Water reward	–	Awareness of water reward	IT, nIT
II	Instrumental training	1	FP and HP presses rewarded	IT, nIT
		2	Press w/HP from any position	
		3	Press w/HP from starting stance	
		4	Single motion press	
III	Cue training	1	Introduce cue 1 coincident	IT, nIT
		2	Lights out condition	IT, nIT
		3	Profiency I	
		4	Introduce cue 2 at median press duration until 1.5 s	IT only
		5	Proficiency II	IT only
IV	Cue choice training	1	Randomized 1:1 cues	IT only
V	Implantation	–	Proficiency III	IT, nIT

### Multiple, single neuron recording

All surgical procedures were carried out under aseptic conditions. Once animals were trained to proficiency, they were provided *ad libitum* access to water for 72 h prior to the surgery to promote hydration and weight gain. Animals were implanted bilaterally with 4 × 4 arrays of Teflon-insulated microelectrodes (0.1–0.7 MΩ; Neurolinc Inc., Briar Ridge, NJ) into the HLSMC (−0.5 to −2.75 AP, −1 to −3.5 ML; Figure 1C) using standard methods in our lab (Moxon et al., 2008). Each array was lowered until the infragranular layer of the cortex was reached, approximately 1.3 mm from the surface of the brain, typically characterized by the activity of large amplitude layer V–VI pyramidal neurons.

Following electrode implantation and post-surgical recovery, animals were returned to normal water restriction. Animals were run for at least three sessions in the behavioral chamber to refamiliarize themselves with the task, after which neural recordings were made while the animals performed the task. On a recording day, animals were lightly anesthetized with 3% isoflurane and 20x VLSI headstages (Plexon, Inc., Dallas, TX) were connected to the electrode connectors at the animal's head. After recovery from anesthesia, the headstages were then connected to the pre-amplifier and sent to the Multichannel Acquisition Processor (MAP, Plexon Inc.) system where they are then digitized and stored at 40 kHz on a host PC.

Single neurons were discriminated using a combination of a real time principal component analysis algorithm and template matching procedures. Multiple single neurons on a single channel were isolated using clustering of waveform projections onto principal component space and auditory verification through a pair of speakers. Once the signals from all 32 implanted electrodes were sorted and verified, the animal was retested in the task either under the nIT or IT condition while neural activity was passively recorded.

## Data analysis

Following a recording session, all behavioral data was post-processed and trials were sorted in Matlab (The Mathworks, Natick, MA). In both nIT and IT groups, animals made short and long duration presses. Typically, a trial press <1 s was considered short, and between 1.5 and 2.5 s was considered long (see Figure 1B, for typical amplitude sensor traces of each press duration). All other trials were not considered in the analysis. Subsets of press duration distributions for each group (Figure 3A—gray, original distributions) were removed in order for the distribution of short and long duration presses to be the same between groups. Analyses were carried out on both the full data sets and the reduced data sets with no effect on statistical significance (refer to Figure 3A).

### Neuronal response properties

While there are many important aspects to the encoding of lever press produced by these animals, here we are strictly interested in how temporal scaling occurs in the HLSMC and whether animals that are not trained to produce particular durations would show this scaling. To accomplish this, single neuron spike times and event times (e.g., times of cue, reward, and amplitude sensor data) were saved while the animals performed either the nIT or IT task. Perievent time histograms (PETHs—the binned spike counts of a given neuron over a large number of trials) of spike times for all channels were then generated (−1.5 s pre, 3 s post window) around the start of press from the amplitude trace (Figure 1B).

To determine if the firing rate of the cells were modulated during the task, the PETHs were smoothed using a zero-phase distortion moving average filter of length 5 bins (25 ms). If the firing rate exceeded the 99% confidence interval of the background average rate, defined as the mean firing rate 1500–1000 ms before press onset, for at least three bins in the unsmoothed response window, the cell was considered to respond significantly to the task. Only cells with a significant response were further analyzed. The following parameters were extracted from the PETHs: (a) Response Magnitude (RM): the background-subtracted sum of the spikes in all the bins in the response window divided by the total number of trials. (b) Peak Response (PR): the bin with the maximum number of background subtracted spikes divided by the total number of trials. (c) First bin latency (FBL): The latency of the first bin that crosses the threshold with respect to the start of press. (d) Last bin latency (LBL): The latency of the last bin that crossed the threshold. (e) Peak latency (PL): The latency of the peak bin, as in Figure 1D. FBL and LBL measures were derived from the smoothed PETHs. The RM, PR, and PL were derived from the unsmoothed PETHs. The duration of the response was defined as the time between the first and last bin latencies. Differences in these parameters were assessed using a one-way analysis of variance (ANOVA) for press type with two levels: short press and long press.

### Identification of climbing activity

A primary indicator of elapsed interval time is the characteristic climbing (or descending) pattern of activity, in which a neuron will either increase or decrease its firing rate nearly monotonically over a given interval (Durstewitz, 2004; Lebedev et al., 2008) separated by two relevant events. Thus, to test the effect of behavioral training on the encoding of temporal intervals by neurons within the infragranular layer of the HLSMC of IT and nIT group rats, we evaluated the firing rate profile for each recorded cell using its PETH for the presence of climbing or descending activity (Figure 1E). Neurons whose firing rates increased or decreased in a window between press onset (as measured by the amplitude sensor) and the mean press duration for a given press type, were considered potentially modulated in a climbing- or descending-like fashion. Climbing activity was confirmed by a linear regression analysis of the change in firing rates (sampled using 5-ms bins, smoothed over a 50-ms window with a zero-phase distortion filter). Neurons with significant task-related modulations (normalized change in firing rate greater than the 90% CI and *p* < 0.001, F-statistic for linear regression) and a coefficient of determination (*R*^2^) greater than 0.25 were considered climbing cells. The sign of the rate of change, further distinguished ascending from descending patterns. Moreover, cells that displayed climbing activity for both long and short press types were distinguished from those who only displayed the behavior during one press type. Neurons that did not meet the selection criteria were considered non-climbing.

### Temporal response parameters

To determine if the climbing activity displayed temporal scaling, the temporal parameters (firing rate slope, normalized change in firing rate, and *R*^2^) from the PETHs for each neuron were compared between short and long duration presses. A hallmark of temporal scaling is that the rate of change of the firing rate increases (or decreases) proportional to the estimated interval (Figure 1F; Gibbon, 1977; Renoult et al., 2006). Thus, temporal scaling reflects elasticity in the neuron's firing rate. Therefore, if press durations are encoded by temporal scaling, the slopes of the regression should be different, on average, for the different durations being estimated while the overall change in firing rate (threshold for action) should remain constant. Because it is likely that there are multiple timing signals in the brain (Merchant et al., 2011), in addition to assessing temporal scaling on trial averaged PETHs, we investigated the role of the peak firing rate of the climbing activity, rather than slope, as a means of temporal accumulation.

### Single trial decoding of temporal intervals

Two separate techniques were used to decode press durations on a single trial basis: a multiple linear regression technique (Carmena et al., 2003; Lebedev et al., 2008) known as Wiener filtering, and an interspike interval histogram (ISIh)-based classification scheme based on our previous peristimulus time histogram (PSTH) based classifier (Foffani and Moxon, 2004). Together, these two techniques allow for a parametric estimation of elapsed time (Wiener filter) and a classification of press type (short vs. long) on a single trial basis.

**Figure 2:**
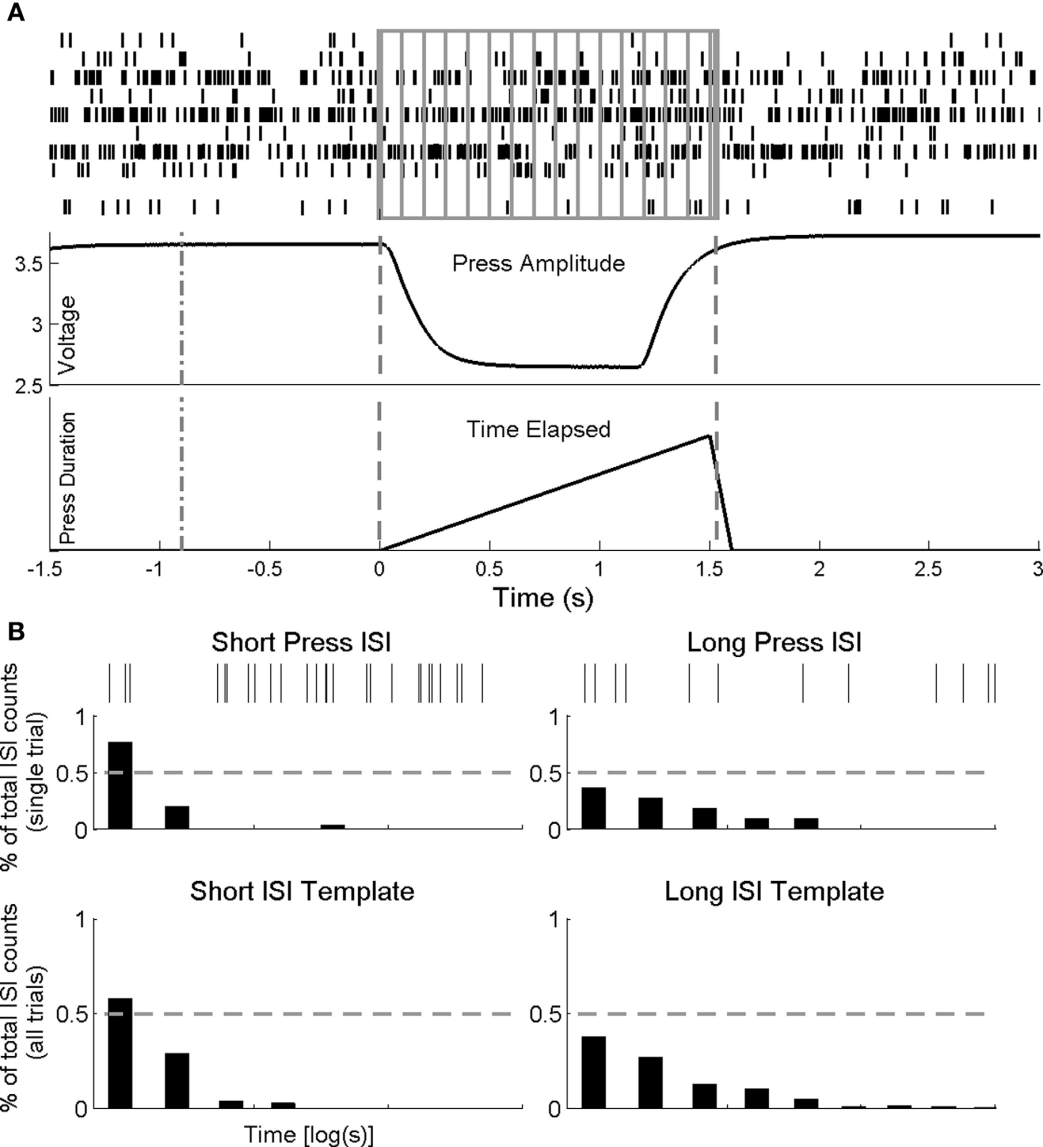
**Single-trial decoding methods**. **(A)** Weiner filter. Spike times (top panel) for recorded neurons (one row per neuron) during a single pedal press (middle trace—amplitude of press; bottom trace—duration of press) are binned at 100 ms and provide inputs into a linear model to predict press duration (see details of the Weiner filter in Methods). **(B)** Interspike interval classifier. Interspike timing during the first 1000 ms from start of press is used to classify press duration. Spike times (top panel) for a single cell during a short and long press and its ISI histograms for the same short (left) and long (right) (middle panel). The single trial ISI's are compared, bin by bin, to templates generated from all remaining short and long duration presses (bottom panel). The template for which the comparison shows the minimum error between each single trial and each template is predicted classification result. If the predicted classification template indicated the correct press duration, then the trial was correctly classified (true positive).

The Wiener filter used in this analysis takes the form

$$D(t)=b+\sum_{\tau =t_{0}}^{t_{f}}w(\tau )z(t+\tau )+\varepsilon (\tau )$$

where ***z***(*t* + τ) is a vector of firing rates at time *t* and time lag τ (where τ is a time of past firing rates), *D*(*t*) is the estimated press duration, b is the y-intercept of the regression model, and ***w***(τ) is the vector of linear weights for each neuron at time-lag τ. For this analysis, 10 time lags preceding time *t*, spaced at 100 ms were used to train and test the filter (see Figure 2A). The filter was trained on the time elapsed since start of press *D*_*P*_(*t*). Predictions of temporal intervals are derived from both the firing rate (per sampled bin) and the rate of change of the firing rate. To train the filter, the linear model takes the neuronal ensemble firing rates in each bin at time *t*, and calculates a vector of weighted values for each neuron and lag in the time window *t* − τ to *t* using the identity

$$w={\left[zz^{T}\right]}^{-1}[D_{P}z^{T}]$$

where ***D*_*P*_** is a vector of current press duration at time *t*. For each recording day, the filter was trained on half of the total number of both long and short presses, and the remainder of the data were used as the test data set. The number of bins and bin size (30 bins, 100 ms bin size) within the window for the training and test data sets were chosen empirically and the results did not change qualitatively with a slightly increased or decreased number of bins or bin size.

To measure the overall prediction accuracy of the filters, two measures were employed: first, the overall correlation coefficient, *R*, between the actual intervals and the predicted intervals, is a widely used measure in neuronal decoding and provides an overall measure of the accuracy of the filter. Second, a linear regression of the predicted interval per trial was computed and a 95% confidence interval around the regression was constructed. If the actual value of the temporal interval falls within this confidence interval, the final predicted temporal interval was considered a True Positive (TP). If, however, the final value was not included in the computed confidence interval, the predicted trial was a False Positive (FP). For a given day then, half of the trials were randomly selected to train the filter and the remaining trials are used to test. This process was repeated 10 times and the average overall *R* (over all trials), and TP and FP rates were tabulated. In this way, TPs and FPs were calculated for all short and long trials independently to assess any differences in single-trial encoding of short vs. long temporal intervals.

As a comparison to the time binning used in this analysis, a separate filter was trained using the total spike count within the overall trial window. If the bin-by-bin firing rate (and thus the slope or rate of change of the firing rate) accounted for by the Wiener filter predicts temporal intervals more accurately than a pure rate coded filter, it suggests that spike timing, and specifically the temporal modulations of climbing and descending neurons, are important to the encoding of temporal intervals compared to the overall firing rate.

In contrast to the parametric output of the Wiener filter, we used interspike timing of single neurons to classify press type (short and long) on a single trial. Because we hypothesize that press duration is encoded through patterns of climbing and descending activity, in which the slope of it firing rate varies proportional to the interval being estimated, the interspike intervals of these neurons should be dissimilar so long as the time between durations is enough to account for significant change in the slope of the activity (spike timing). Thus, if this activity is encoding press duration, there should be sufficient information about the press duration within the first second of each interval to classify on a single trial.

The ISIh based classifier is an extension to our PSTH based classifier (Foffani and Moxon, 2004) in which trial averaged peristimulus activity serves as a template to discriminate neuronal responses on a single trial basis. For each recording session, trials were classified singularly by generating the trial average ISIh for both press types for all remaining trials (leave one out) using only the first 1 s of spiking following the start of press. ISI histograms were calculated by first determining interspike timing for all trials and neurons and then binning the interspike times across trials (50 ms bin size) with each bin represented as the proportion of counts from the total interspike count. Once the templates (Figure 2B) were calculated, the trial ISIh was calculated with the same bin size. The trial was then compared bin-by-bin to both short and long ISIh templates, and the template that had the minimum comparison error (quantified as the sum of the square error between each bin in the templates and trial ISI histograms) was the classification result. This was performed on every cell in the ensemble; the greatest proportion of neurons with the same classification result was the overall classifier output. Performance of the classifier was the quantified by totaling the number of correct classifications (i.e., classified short when single trial was short) and dividing by the total number of trials classified for each press type. We determined chance level for the classifier using a bootstrapping technique on ISIh templates computed from randomly interspersed short and long duration trials. By repeating this procedure over a large number of iterations (bootstraps = 30) we calculated group average chance levels (short chance = 47.5%, long chance = 48.1%).

### Statistics

For all statistical tests, we investigated within group differences in neural activity between short and long duration presses. To compare neuronal response properties [RM, LAT(latency), etc.], temporal parameters (*R*^2^, ΔFR, and slope) and decoding performance a multivariate one-way ANOVA with a Bonferroni's multiple comparison *post-hoc* test (non-directional, Bonferroni corrected significance, *p* = 0.0071) was used. In the case of unequal sample sizes, bootstrapping tests (100 boostraps) were performed using randomized subsets of the data to ensure there was no effect of sample size on the significance levels of the results. All numerical values are presented as mean values ± S.E.M. unless otherwise specified.

## Results

### Behavioral performance

**Figure 3:**
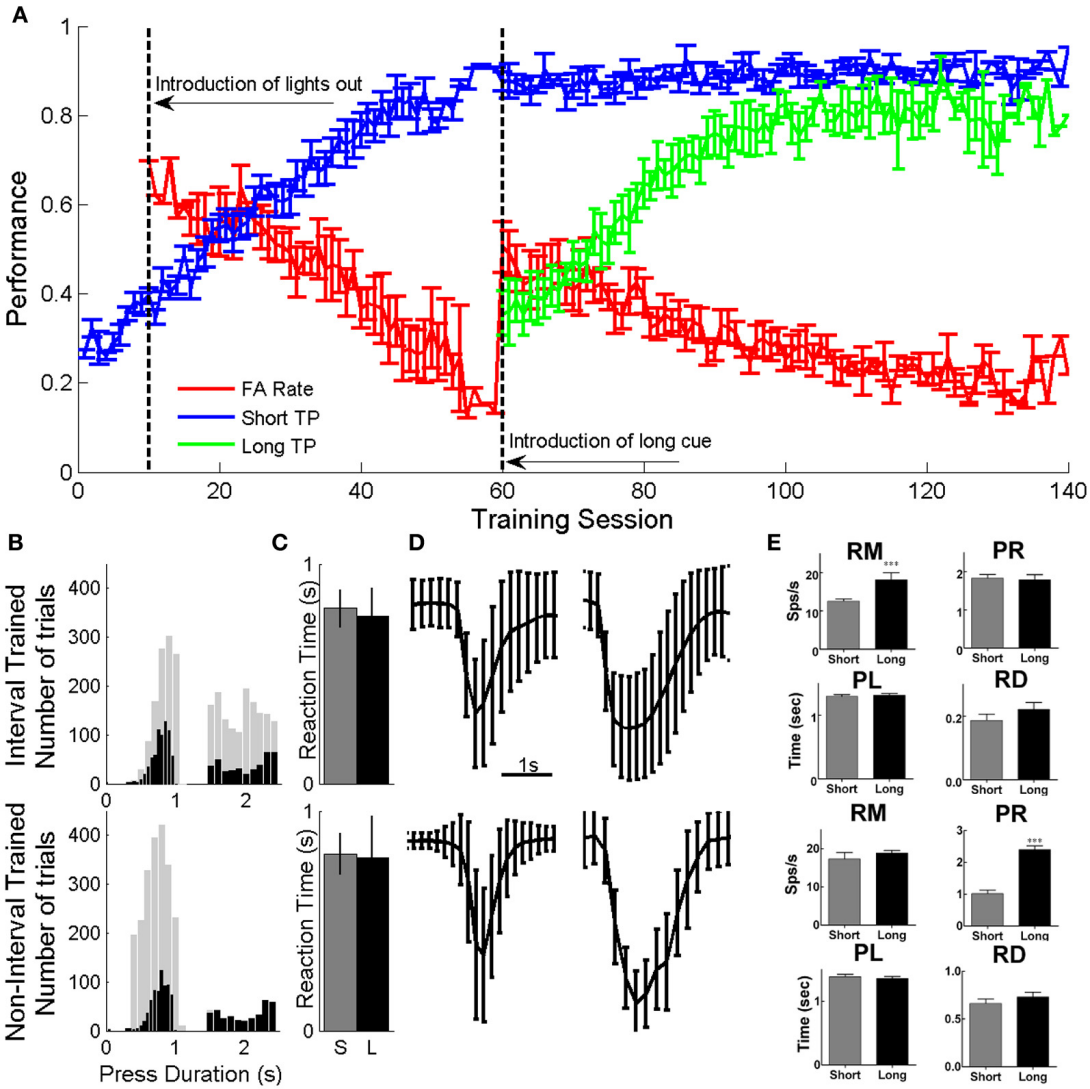
**Behavior and neural responses during the task**. **(A)** Behavioral performance during phases of training expressed as the number of correct presses out of the total number of cues given. Animals gradually learned to associate short and long duration cues with short (blue) and long (green) duration presses, while, decreasing the rate at which spurious presses (False alarms—red) are made within 2 s of cue presentation. A subset of these data were previously presented in Knudsen et al. (2011). **(B)** Original (gray) and reduced (black) press duration distributions for IT (top) and nIT (bottom) groups. Trials were removed from each group to allow similar distributions of short and long presses for comparison between groups. **(C)** There were no differences in average reaction time between groups or between press durations. **(D)** Average amplitude traces from reduced press durations from **B**. There were no differences between groups in the durations or amplitudes of the presses across groups for short or long presses. **(E)** Differences in neuronal response properties. RM, response magnitude; PR, peak response; PL, peak latency; and RD, response duration for IT (top) and nIT (bottom) animals between short and long duration presses. For IT animals, short and long duration presses were significantly different in the magnitude of the response, while only peak response was significantly different for nIT animals. This suggests that the encoding strategies for different press durations are different between the two groups. ^***^p < 0.001.

Both groups of animals were able to perform a single press with minimal requirements within a similar number of training session (81.2 ± 1.7 for nIT and 58.4 ± 2.4 for IT). To become proficient in both short and long presses, the IT group required an additional 84.6 ± 3.6 (average of 143 ± 2.32 total) training sessions, while (Figure 3A—IT group). The distribution of press durations during neural recording sessions for IT animals was different from those of nIT animals. nIT animals tended to make shorter duration presses while IT animals had an even distribution of short and long duration presses. While it is expected that the histograms should be mostly bimodal according to Gibbon's scalar property theory of behavioral timing (Gibbon, 1977; Gibbon et al., 1997), there is a distinct trimodality to the distribution of long duration presses in the IT group. For the animals tested here, there were two subsets of animals in the IT group: one that tended to make presses with a mean duration of approximately 2.3 s (*n* = 2) and one that made presses with a mean duration of approximately 1.7 s (*n* = 4); both mean times were within the range of values for accepted long duration presses. Due to unequal numbers of trials between both groups, it was necessary to exclude trials from each group (see “*Methods*”) to have similar distribution of trials across groups. For both groups, the durations of presses ranged from approximately as short as 0.4 s to as long as 3 s (Figure 3B—gray bars). We selected short presses that ranged from 0.4 to 1 s (IT: mean 0.8914 ± 0.132 s; nIT: mean 0.76 ± 0.097 s) while for long the range was 1.5–2.5 s (IT: mean 2.001 ± 0.29 s; nIT: mean 2.049 ± 0.017 s) as shown in Figure 3B (black bars). There were no differences in mean reaction times between the two groups for either short or long press durations (Figure 3C). Average amplitude traces for both short and long presses for both IT and nIT animals (adjusted distributions) are shown in Figure 3D. Given this subset of presses, there were no differences between the distribution of short (unpaired *t*-test, *p* = 0.178) or long (unpaired *t*-test, *p* = 0.972) duration presses between groups.

### Neuronal responses properties

Neuronal responses varied between short and long duration presses differently for IT and nIT animals (Figure 3E; Table 2). For IT animals, the magnitude of the response was significantly different between short and long presses (*p* = 0.0012). The peak of the response, FBL, LBL, and PL were not significantly different. For nIT animals only the average peak of the response was different between short and long presses (*p* < 0.001). RM, FBL, LBL, and PL were not significantly different. These results suggest differences in the encoding of press duration in nIT vs. IT animals; in IT animals this is reflected by difference in the magnitude of the response, while in nIT animals this is reflected by differences in the peak of the response.

**Table 2:** **Comparison of neuronal response properties between the short and long press for interval trained and non-interval trained animals**.

	**Interval trained**		**Not-interval trained**	
	**Short press**	**Long press**	**Short press**	**Long press**
Response magnitude (spikes/s)	***12.57* ± *0.578***	***18.13* ± *1.866***	*17.31* ± *1.647*	*18.89* ± *0.660*
Peak response (spikes/s)	*1.832* ± *0.092*	*1.787* ± *0.133*	***1.02* ± *0.109***	***2.402* ± *0.115***
First bin latency (s)	*1.0996* ± *0.136*	*1.1462* ± *0.03*	*1.095* ± *0.039*	*1.002* ± *0.046*
Last bin latency (s)	*1.3228* ± *0.134*	*1.4055* ± *0.032*	*1.759* ± *0.046*	*1.734* ± *0.994*
Peak latency (s)	*1.256* ± *0.029*	*1.313* ± *0.027*	*1.386* ± *0.037*	*1.358* ± *0.032*

For interval trained animals, bolded response magnitude indicates differences between short and long press at p < 0.005. For non-interval trained animals, bolded peak response indicates differences between short and long press at p < 0.001.

### Evidence of climbing activity for both non-interval and interval trained animals

**Figure 4:**
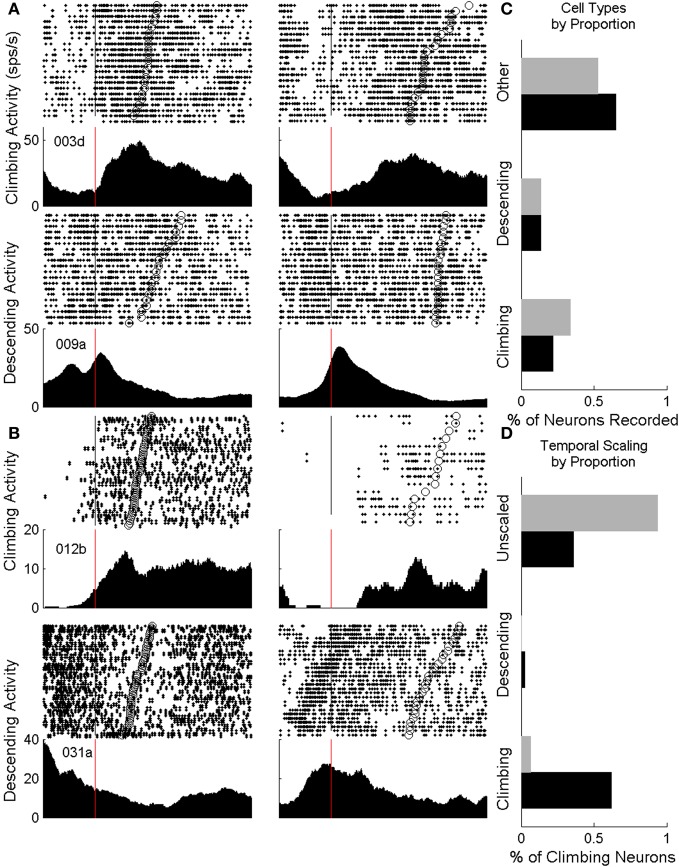
**Neurons from both groups displaying climbing activity**. **(A)** Climbing and descending cells for IT animals. The perievent response peaks (troughs) at the end of press (open circles, rasters) while the overall change in firing rate stays the same between press durations (left: short, right: long duration). Vertical lines denote the start of press. **(B)** Identified climbing and descending cells for nIT animals display similar patterns of activity, though in a less conserved manner between short and long durations. **(C)** Proportions of identified climbing cells (ascending and descending) neurons are roughly equivalent between IT (black bars) and nIT animals (gray bars). **(D)** When assessing neurons for temporally-scaled climbing activity, over 50% of IT neurons were temporally-scaled (black bars), while fewer than 10% were temporally scaled in nIT animals (gray bars).

All animals from both the nIT and IT groups had neurons that displayed climbing and descending activity. Rasters and perievent trial histograms for four neurons recorded during the task (2 IT cells, 2 nIT cells) are shown in Figures 4A,B. Of these, two of the neurons shown exhibit climbing firing rates, while 2 show descending rates. The perievent responses represent the smoothed activity of neurons over many trials and do not reflect the trial by trial variability of the neurons' firing rates. However, on average for climbing type activity, cells begin to increase their firing rates steadily from the time of press onset (Figures 4A,B, red lines) until the time of press release (Figures 4A,B, open circles on rasters) at which point they peak and begin to decrease their rates. Conversely for descending activity neurons peak at or near the time of press onset (red lines) and begin to decrease their firing rates at a rate (slope) proportional to the interval being estimated until the firing rate begins to climb or levels off.

In the rasters, the density of spike times on single trials reflects the trial-average activity and the periods of increased firing correspond well to the duration of press qualitatively. In some cases there is a strong upward modulation in activity before the endpoint start of press as the animal prepares and initiates its movement, regardless of whether the cell shows climbing or descending modulation patterns, suggesting multimodal encoding by these neurons (Wang et al., 2007). Interestingly, this pre-start of press activity is not always reflected by the same neuron between press durations, however, it could reflect the timing of the animals' response initiation. Long duration presses in nIT animals are qualitatively less consistent in the smoothness of the rate of climbing or descending firing rate compared to IT animals.

Quantitatively, for IT animals (*n* = 6, 62 recording sessions, 3377 neurons) 21.7% of neurons showed temporal profiles consistent with climbing activity (*R*^2^ > 0.25, ΔFR > 2, positive slope) for short, long, or both press durations, while 13.4% displayed descending activity (negative slope) (Figure 4C). Similarly, climbing and descending activity was found for all animals in the nIT group (*n* = 5, 40 recording sessions, 1690 neurons). Of all recorded neurons, 33.8% met the criteria for climbing activity for at least one duration press, and 13.4% met criteria for descending activity (Figure 4C). Therefore, regardless of task, climbing and descending activity takes place in roughly the same proportion of recorded neurons.

### Climbing and descending activity is temporally scaled in IT but not nIT animals

Despite the fact that IT and nIT animals displayed similar distributions of climbing and descending neurons, only neural activity recorded from animals performing the IT task displayed temporal scaling. Temporal scaling, wherein the rate at which a neuron's firing rate increases or decreases changes proportional to the estimated interval while the overall threshold for action the firing rate must reach signifying the end of the estimated interval (change in firing rate), is one of the primary characteristics of temporal estimation processes and has been widely observed in the literature. Therefore, for the recorded climbing and descending activity to encode press duration through the rate of climbing activity, this activity must vary its slope during the press periods of the behavior in a manner temporally scaled to the duration of press.

**Figure 5:**
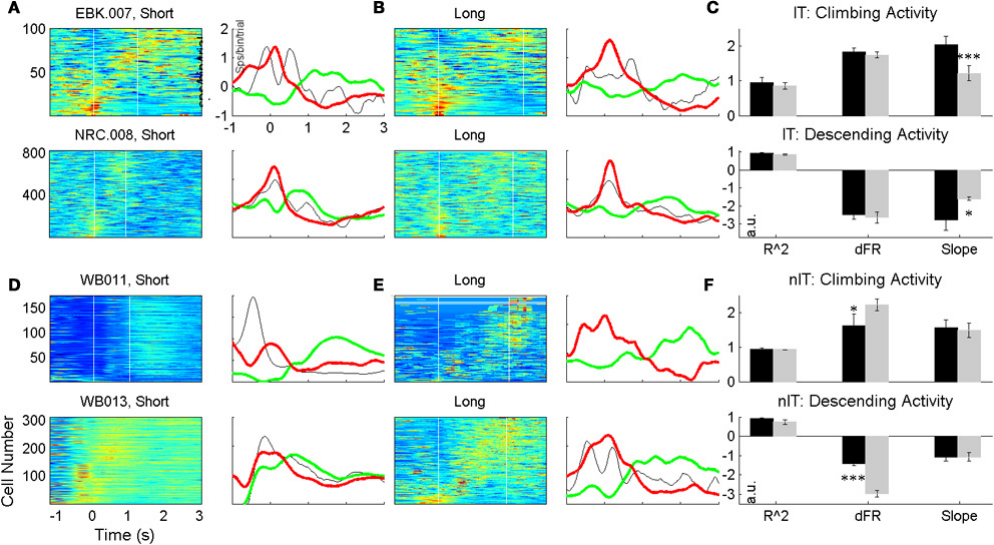
**Trial averaged ensemble modulations during short (A,D) and long (B,E) duration presses for IT (A–C) and nIT (D–F) animals**. Average traces of climbing (green), descending (red), and other (black) activity shown to the right of each pseudocolor image. Qualitatively, slopes between press durations for IT animals seem different, while appear more similar for nIT. Evaluation of R2, change in firing rate (dFR) and slope reveal population differences in the encoding of press duration through climbing and descending activity **(C,F)**; while IT animals encode duration through temporal scaling of climbing activity, nIT animals do not. ^*^p < 0.05, ^***^p < 0.001.

Figure 5 displays color-coded PETHs for all neurons showing climbing or descending activity for at least one press type across days for two representative animals from both the IT and nIT groups. In these plots, to account for differences in overall firing statistics between individual neurons, all cells were normalized by subtracting the mean of the firing rate and dividing by the SD (for each press period). The neurons were then ranked according to their normalized rate changes. Cells with the strongest climbing activity are shown at the top of each panel, the strongest descending modulations are at the bottom, and cells with modulation unrelated to climbing or descending activity patterns are shown in the middle. For each subpopulation of cells, the average patterns of activity are plotted (Figure 5; green—average climbing cells, red—average descending cells, and black—average cells modulated in other ways). Cells from both IT and nIT groups exhibit on average patterns of climbing and descending activity for both press durations, however, features of the trial average rates relevant to temporal scaling (slope and change in firing rate, Figure 1F) appear less conserved in nIT animals compared to IT between short and long duration presses.

To quantitatively assess the temporal scaling properties of climbing and descending activity, we calculated the average slopes, *R*^2^s and normalized change in firing rate for all neurons displaying climbing and descending modulations during the task, shown in Figures 5C,D. For both groups, there were no significant differences between *R*^2^ values (IT: *t* = 0.4961 for climbing, *t* = 0.1756 descending; nIT: *t* = 0.0206 climbing, *t* = 0.6242 descending). In IT animals, consistent with temporal scaling, the normalized change in firing rate was not different between press durations (IT: *t* = 0.3733 climbing, *t* = 0.3674 descending), however, there were significant differences in nIT animals (nIT: *t* = 3.501, *p* < 0.005 climbing, *t* = 5.699, *p* < 0.0001, descending—see Table 3 for summary of values). Therefore, the threshold for action for the IT animals' climbing neurons was not different between press durations while there was no consistent threshold for the nIT animals' climbing neurons during different press durations. Importantly, in IT animals the slopes for short presses were significantly greater than long for both climbing and descending modulated neurons (IT: *t* = 3.817, *p* < 0.0005 climbing, *t*_(12)_ = 2.82, *p* < 0.005 descending), compared to no temporal scaling of slope for nIT group neurons (nIT: *t* = 0.2626 climbing, *t* = 0.0652 descending). Thus, on average, climbing cells within the HLSMC of rats trained to produce two distinct durations of hindlimb press (IT) utilized temporal scaling to encode the estimation of press duration while climbing cells from nIT animals appear to encode information about press duration in the peak of the response.

**Table 3:** **Comparison of climbing activity between short and long press for interval trained and non-interval trained animals**.

	**Interval trained**		**Not-interval trained**	
	**Short press**	**Long press**	**Short press**	**Long press**
**CLIMBING**				
*R*^2^	*0.958* ± *0.5*	*0.851* ± *0.093*	*0.934* ± *0.033*	*0.929* ± *0.015*
ΔFR	*1.825* ± *0.121*	*1.745* ± *0.0865*	***1.628* ± *0.336***	***2.226* ± *0.173***
Slope	***2.039* ± *0.242***	***1.219* ± *0.222***	*1.558* ± *0.235*	*1.483* ± *0.212*
**DESCENDING**				
*R*^2^	*0.9411* ± *0.024*	*0.868* ± *0.0248*	*0.906* ± *0.043*	*0.733* ± *0.114*
ΔFR	−*2.5* ± *0.247*	−*2.652* ± *0.322*	**−*1.404* ± *0.114***	**−*2.986* ± *0.291***
Slope	**−*2.773* ± *0.587***	**−*1.6* ± *0.102***	−*1.085* ± *0.204*	−*1.067* ± *0.222*

For interval trained animals the bolded slope of the response for both climbing (*p* < 0.001) and descending (*p* < 0.05) cells was significantly greater during the short press compared to that during the long press. For non-interval trained animals, the bolded change in firing rate for both climbing (*p* < 0.05) and descending (*p* < 0.001) cells was significantly greater for long presses compared to that of long presses.

We then investigated the proportion of neurons that showed temporal scaling out of the total population of neurons exhibiting climbing behavior. For IT animals, of the population of neurons that showed climbing activity (733 of 3377 neurons), 53.2% (390 neurons) displayed temporally scaled climbing activity and 2.3% of descending neurons (17 neurons) were temporally scaled. While 33.8% of neurons (572 of 1690 neurons) recorded from nIT animals met criteria for climbing activity, only a small proportion of this population (6.47%; 37 neurons) displayed temporally scaled climbing activity, while no neurons displayed temporally scaled descending patterns of activity (Figure 4D). We further investigated the PRs of climbing neurons in the both groups as an alternate means of temporal coding (Merchant et al., 2011). Similar to the results above, over all trials, 190 of 572 nIT climbing neurons (33.9%) had significantly different peak amplitudes with the same rates of climbing (*t* = 15.188, *p* < 0.0001 for peak; *t* = 1.882, *p* = 0.0615 for slope). In the IT group there was a small subpopulation of climbing neurons that also appear to encode press duration through PR with no temporal scaling (47 of 733 neurons, 6.41%, *t* = 7.137, *p* < 0.0001 for peak; *t* = 1.742, *p* = 0.0848 for slope). Thus, although, there are proportions of climbing neurons in both tasks that peak in firing at different rates for the duration of press, the proportion of these neurons is greater when temporal scaling mechanisms are not present.

### Wiener filter predicts temporal intervals for IT but not nIT animals

**Figure 6:**
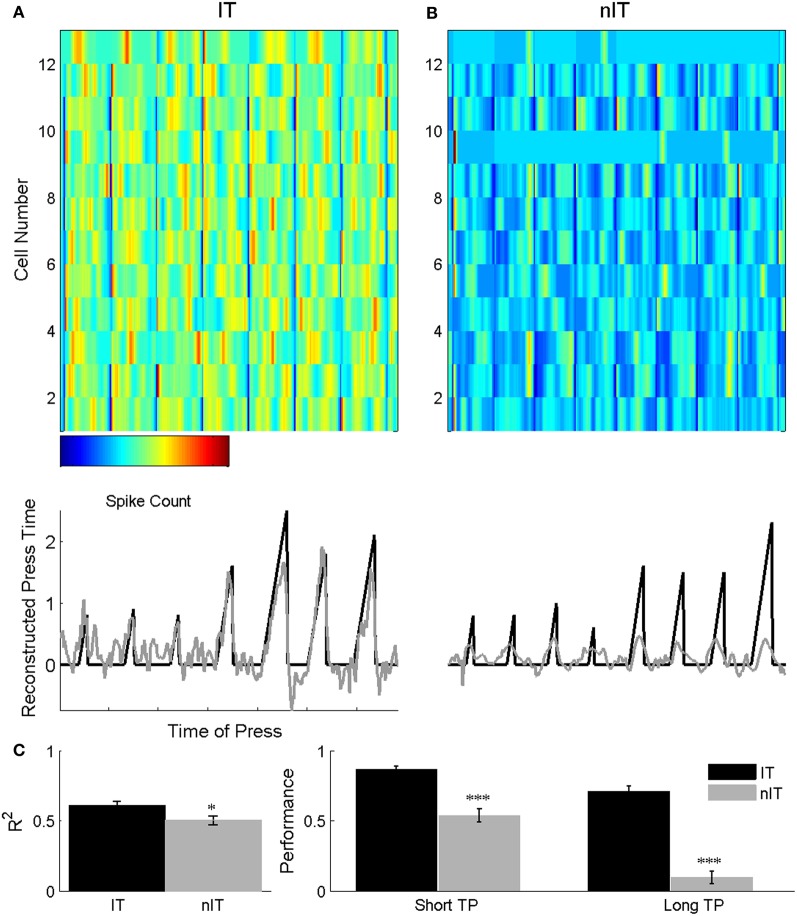
**Example ensemble activity (A, B top) used to reconstruct press duration (bottom) for an IT (A) and an nIT (B) animal**. IT neurons are clearly modulated throughout short (**A**, left of panel) and long (**A**, right of panel) duration presses, while nIT neurons appear strongly modulated around the start and end of press, but not throughout the press period. In the reconstructions (middle), solid black lines are actual press durations and gray lines are predicted durations from the ensemble activity. IT ensemble activity faithfully reproduces press duration while nIT activity predicts press periods but consistently underestimates press duration. Time axes for ensemble activity and reconstructions are the same. **(C)** Overall *R* values for Wiener filter reconstructions for IT are significantly greater than nIT (unpaired *t*-test, *p* < 0.05). When using the error between actual and predicted press duration to quantify decoder performance, the filter for the IT group significantly outperforms the nIT filter on short and long duration presses. ^*^p < 0.05, ^***^p < 0.001.

Population activity on single trials from IT animals predicted press duration significantly better than nIT population activity using a Wiener filter designed to take advantage of temporal scaling (Lebedev et al., 2008). Examples of press duration predicted from ensemble activity and actual press durations from one IT and one nIT animal are shown in Figure 6. In both cases, the combined noisy ensemble activity (Figure 6A, top) contributes to the prediction of press durations (Figure 6A; gray lines), which can be used to reconstruct the actual press durations (Figure 6A, middle; solid black lines). For each successive trial the neural activity of IT animals is clearly modulated throughout the duration of press, which translates into accurate predictions of press duration. Conversely, nIT animals' firing rates are modulated around the start and end of press and as a result, it consistently underestimates longer press durations.

Using all neurons in the ensemble to train and test the filter in a leave-on-out approach, correlations for the IT group approach values seen in other studies (Carmena et al., 2003; Lebedev et al., 2008) for predicted press duration (*R* = 0.61 ± 0.03), whereas the overall correlations for the nIT group were significantly lower (*R* = 0.508 ± 0.031; unpaired *t* test, *p* = 0.048). To quantify the performance of the filter on a single trial basis, we computed a linear fit of each single trial and constructed a 95% confidence interval around the fit. With this metric, the Wiener filter for IT animals predicted 86.5 ± 2.63% of short presses and 71.2 ± 3.71 of long presses, while the filter was able to predict only 54.1 ± 4.69% of short presses and 10.3 ± 4.42% of long presses for the nIT group (10 bootstraps, IT chance: short = 17.8%; long = 3.83%; nIT chance: short = 29.19%, long = 0.0836%). As expected, overall performance when we trained a separate filter using the total spike count within the trial window was poor for both groups (IT: *R* = 0.221 ± 0.017; nIT: *R* = 0.277 ± 0.023). This control serves as a verification of the role of spike timing vs. spike count on a single trial basis and suggests that for both groups of animals, spike timing during behavior is more important than overall firing rate when attempting to decode parametric press duration.

### ISIh classifier discriminates press duration for IT but not nIT animals

**Figure 7:**
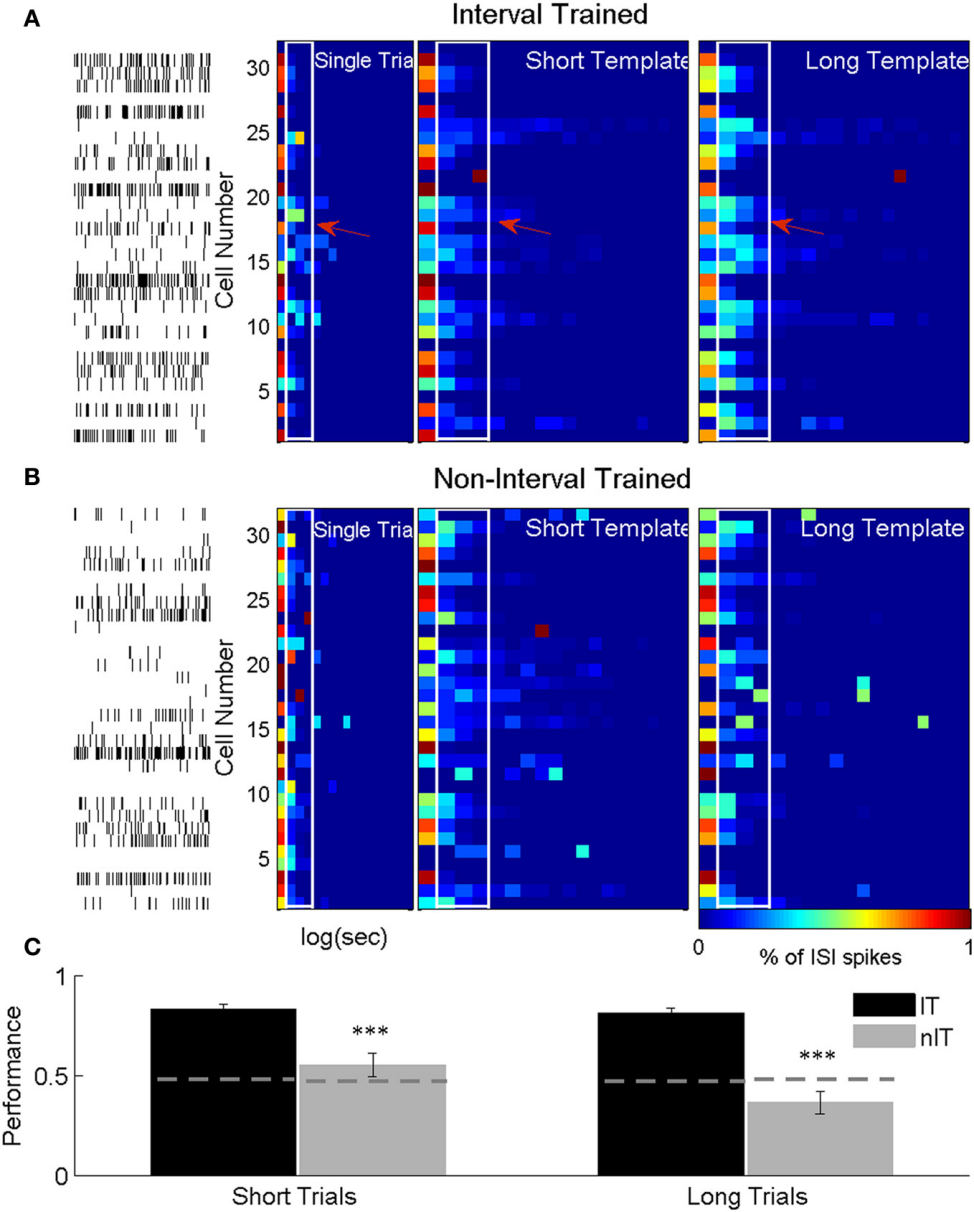
**ISIh-based classification press duration**. **(A)** ISIh-based classification of single short duration trial for an IT animal. **(B)** ISIh-based classification of single short duration trial for a nIT animal. For both **(A)** and **(B)**, the left most panel are the single neuron spike times (one neuron per row) the single short duration trial. The next panel is the binned ISI histograms for the ensemble firing (normalized to the peak bin) for the same trial. The next two panels show the average population ISI histograms for either all of the remaining the short trials or all of the long trials (right most panel) trials. These population histograms serve as templates against which to compare the single trial. Distinct differences between the short and long templates can be seen for interval trained animals (white outlines with arrows), while there are fewer differences between templates in nIT. **(C)** Because of this lack of differences, both short and long trials are not well classified for nIT animals compared to IT animals. Thus, interspike timing for IT animals results in significantly better classification performance for both short and long duration trials compared to nIT animals. ^***^p < 0.001.

Using our ISIh based classifier we were able to achieve significantly higher decoding accuracy for IT animals compared to nIT animals. Examples of classification of single short duration presses for both groups are shown in Figures 7A,B. In each, single trial rasters for each neuron in the ensemble and the resultant trial ISIh are compared to trial average ISIh templates for short and long duration presses, represented as pseudocolor images. In the IT example, marked differences between short and long duration templates allow for clear discrimination of the single trial (Figure 7A). However, in the nIT example, the templates are qualitatively more similar, which leads to uncertainty in the discrimination of single trials (Figure 7B), a reflection of similar temporal profiles of neurons within the first second of press onset during both short and long duration trials.

Across all recording sessions for IT animals, the ISIh based classifier correctly classified 83.1% (SEM: ±2.63%) of short duration trials as short and 81.1% (±2.48%) of long duration trials as long. When nIT data was classified, only 55.4% (±5.83%) and 36.6% (±5.66%) of short and long duration trials, respectively were classified correctly. For IT animals the ISIh based classification performs well above chance levels (short chance = 47.5%, long chance = 48.1%; Figure 7C, dashed gray lines) due to large differences in neuronal responses during long and short trials (Figure 7A), while for the nIT group because there are minimal differences in spiking activity between short and long trials, the classifier fails to perform better than chance, and in fact, performs well under chance values for long duration trials.

## Discussion

### Overview of findings

In the present study we address the question of whether temporal intervals are encoded by infragranular neurons within the HLSMC of rats trained to produce hindlimb presses, and if this encoding differs between animals that were rewarded for presses of a specific duration (IT animals) and animals that were rewarded for presses of any duration (nIT animals). Analysis of the average activity of neurons revealed subpopulations of neurons in both groups that either linearly increased or decreased their firing rate throughout the press period demonstrating that climbing activity, *per se*, is not dependent on an internal estimate of elapsed time. However, these patterns of climbing or descending activity were temporally scaled in IT animals but not in animals who trained to make a press without consideration of the interval. Furthermore, analyses of the temporal profiles of climbing neurons in nIT animals revealed a subpopulation of neurons that reached a peak firing rate well correlated to press duration. These results suggest that the mechanisms of temporal scaling are only engaged when the animal is making an estimation of elapsed time. To further support this conclusion, single trial decoding using parameters of temporal scaling allowed for reasonable decoding accuracy of press duration for IT animals but not nIT animals. Thus, although, time estimation is essential for guiding behavior, the contributions of temporal encoding to the overall spatiotemporal motor commands that generate overt movements are highly contingent upon the context of the movement. Moreover, unlike studies (Roux et al., 2003) where no climbing activity (tonic activity) was observed during delay periods where the delay was uncertain, we observe climbing activity when animals are not explicitly timing press duration (nIT), albeit in a non-temporally scaled, but rather a peak rate-scaled manner. This suggests that patterns of climbing activity exist outside of a temporal estimation context, but only when task demands require estimation and decision making processes do temporally scaled patterns of activity become active.

### Effect of behavioral training on the encoding of temporal intervals

The observed climbing or descending patterns of activity we found in this study are in agreement with what has been observed in the literature in premotor cortex (Romo and Schultz, 1987; Crammond and Kalaska, 2000; Lebedev et al., 2008), supplementary motor area (Mita et al., 2009; Merchant et al., 2011), primary motor cortex (Roux et al., 2003; Lebedev et al., 2008), posterior parietal cortex (Janssen and Shadlen, 2005), prefrontal cortex (Nika and Watanabe, 1979; Goldman-Rakic, 1995; Lebedev et al., 2004), inferotemporal cortex (Reutimann et al., 2004), striatum (Schultz and Romo, 1988; Schultz et al., 1997), and thalamus (Komura et al., 2001). In these previous studies, climbing (or descending) activity was observed during delay period-oriented experimental paradigms in which subjects are asked to discriminate (Roux et al., 2003), estimate (Mita et al., 2009), or generate known (Lebedev et al., 2008) intervals. In many cases (see Janssen and Shadlen, 2005, for saccade task), animals are required to use their forelimbs to generate the behavioral output of timing. Thus, it is well established that areas of the brain devoted to fine motor control (forelimbs, hands) display climbing and descending activity to encode interval time during delay periods. We demonstrate here for the first time that areas of the motor cortex where fine temporal motor control is behaviorally less extensive (i.e., HLSMC) similarly exhibit these patterns of climbing and descending activity, suggesting that this type of activity is a general property of the sensorimotor systems. This result is not altogether surprising. However, it is possible, given that these animals are quadrupeds, that this type of activity would not develop within the HLSMC and that a simple rate code would be sufficient for any task that separated hindlimb function from forelimb function. However, having now established that climbing and descending activity do exist in the HLSMC, we are in a position to further understand how this activity is used to encode temporal intervals.

Importantly, when the animal must make an estimate of the elapsed time in order to make a press of the correct duration and earn a reward (i.e., IT animals), HLSMC neurons whose firing rates climb or descend are temporally scaled. Temporal scalability has been demonstrated in tasks where animals are trained on multiple delays (Komura et al., 2001) or exhibit variability in behavioral timing (Renoult et al., 2006; Lebedev et al., 2008): climbing (descending) activity peaks (troughs) around a threshold that is conserved across multiple durations at a rate proportional to the interval being produced or estimated. Interestingly and unlike previous studies, when the duration of the press is irrelevant and the animals learn only to make a press within a finite window following cue presentation, temporal scaling fails to occur. In this case, differences in press duration are encoded by neurons varying their peak firing rate for different press durations. For IT group animals, the RM is greater for long duration presses than short reflecting differences in the slope of firing rate. For nIT animals there is no difference in RM (similar slopes) though PR is significantly greater for long presses compared to short, which reflects the greater overall change in firing rate for long compared to short duration.

It is likely that nIT animals encode for press duration as a general time accumulation process. This is reflected in the greater PR and larger normalized firing rate for the long duration presses compared to the short duration presses, which could reflect a different timing mechanism when the duration of press is not tied to reward. There are potentially multiple clock signals within the motor cortex during timed behaviors (Merchant et al., 2011). nIT animals' neurons show evidence of unscaled climbing activity during the task on average with no overall change in slope while having significantly larger change in firing rate during long duration presses compared to short. When analyzing climbing activity from nIT animals, a subpopulation of climbing neurons not only had greater peak amplitudes, but they climb at similar rates and peak at similar times. This could reflect an alternative encoding strategy for unplanned or unestimated temporal intervals, i.e., the temporal basis of movement. These strategies could be more prevalent in the nIT group where there is less explicit timing of the behavior (compared to IT animals), in which case we see an increased prevalence of climbing-type neurons compared to IT animals, whose neurons encode duration in a very specialized (e.g., context dependent) manner through temporal scaling.

However, a scaled timing signal is not required to make presses of different durations. Moreover, it is not immediately clear how the scaled timing signal informs the motor command signal. In the context of the IT task, the scaled timing signal peaks at the same threshold, just displaced in time. Therefore, one possibility is that this threshold could be the point at which a motor command (in this case, lift off the pedal) is executed. This threshold could indicate a level at which the amount of input current necessary for neurons involved in signaling the desired movement command is reached and the movement takes place. Alternatively, when the peak of the climbing is linked to press duration (rather than slope), as in nIT animals, this could reflect a more generalized form of timing where rather than a necessary threshold for action, the PR itself encodes for movement time and the downstream motor units act off of this.

There are some limitations to the current study. While both IT and nIT animals were rewarded after a fixed amount of time (IT: 750 ms short press, 500 ms for long press; nIT: 500 ms) after the end of press regardless of press duration, there was no incentive for nIT animals to prolong the press. Hence, a larger proportion of total presses were of short duration in the nIT group compared to the IT group. Additionally, we used video recordings and a variable output amplitude sensor to track animal performance during the task, but perhaps an additional measure of movement such as EMG would allow an analysis of any differences in patterns of activation between different press durations and groups. Despite these limitations, animals made a sufficient number of quantitatively similar presses to evaluate climbing and descending activity across long duration trials as we matched IT and nIT press duration distributions in the analysis and the results did not change from the full IT press distribution to the nIT-matched distribution.

### Ensemble decoding of temporal intervals

To support our conclusion that temporal scaling was only engaged when the duration of press was estimated and not when the duration of press was arbitrary, we used the combined activity of multiple single neurons within the HLSMC to decode press duration on a single trial basis using two methods: the multiple linear regression technique known as the Wiener filter, and a classification scheme based on features of the ISIh. Linear decoding techniques have been utilized to reliably predict parameters of movement kinematics (Carmena et al., 2003) and more recently to predict temporal intervals of monkeys making self-timed button presses (Lebedev et al., 2008). Similar to Lebedev et al. (2008), the Wiener filter used in this study takes bin by bin changes in firing rate (slope) into account to estimate the elapsed press time, and produced good predictions of both short and long duration presses for IT animals. However, unlike this study, the filter failed to perform as well in predicting press durations from nIT group animals that produced long duration presses with no temporal context, consistently underestimating long durations despite similar ranges of press duration. As climbing and descending type activity is well suited to this decoding approach, the overall poor estimation performance for animals where climbing and descending activity is not encoding press duration through temporal scaling, and the lack of single trial differences in firing rate between different durations support our single neuron analysis of an alternate encoding of elapsed time.

To further clarify that different mechanisms are engaged for conscious estimates as opposed to arbitrary durations, the interspike interval was used to decode single trials. This approach takes advantage of the results that the spiking of climbing neurons during different press durations should have distinctly different ISI distributions if they are engaged in temporal scaling to encode the duration. As with the Wiener filter, the ISI achieved better classification performance for IT animals compared to nIT animals. Taken together, the single neuron data and these decoding approaches clarify that temporal scaling is a function of the conscious estimation of time and not in response to elapsed time that is not behaviorally relevant.

### Cognitive differences in the tasks

Clearly the presence or absence of temporally-scaled climbing and descending activity has no overt effect on animal behavioral performance as animals from both IT and nIT groups still made short and long duration presses in response to the GO cue whether their neurons were encoding duration through climbing and descending activity or not. The temporal context of the behavioral task (Roux et al., 2003; Merchant et al., 2011) may be the only contributing factor to differences in temporally scaled climbing and descending activity between groups; when temporal inference or estimation is not required, then temporally-scaled climbing and descending activity does not emerge. It may, however, be that there are additional cognitive processes occurring in the IT hindlimb task compared to the nIT hindlimb task that are worth discussing. Top down executive control of motor action (Narayanan and Laubach, 2006), working memory (Miller et al., 1996), and decision making (Kim and Shadlen, 1999; Rorie et al., 2010) are necessary aspects for successful performance of the IT task in which animals must make a decision about the duration of press to produce based on the cue delivered, and must then estimate the elapsed time during press to ensure it is of sufficient duration for reward. When animals are not required to do this (nIT), the task is reduced to the preparation and execution of an isolated movement (although, over the same time range as IT animals), thus, eliminating the need to estimate the duration of press as it is not relevant to receive reward. It remains unclear what exactly is driving long press behavior in animals not asked to make a long press, however, it seems likely that duration estimation through temporally scaled climbing activity could emerge if the timing of the task were made relevant to nIT animals. Despite the absence of temporally scaled climbing activity, there were HLSMC neurons that did seem to encode some aspect of press duration through peak firing rate, however, this could be a general feature of motor timing that does not require increased cognitive involvement.

As such, it would be beneficial in future studies to further investigate longitudinal effects of behavioral training on the encoding of temporal intervals. Training the animals in the interval training paradigm and subsequently removing all information about required press duration from the cue and rewarding animals for any press may suppress or eliminate any encoding of temporal intervals through temporally scaled climbing and descending activity, or it may persist. If neurons cease to fire in a temporally scaled climbing or descending fashion, then it could be concluded that this type of activity is only present when important in the context of the task. If, however, the activity persists, it would suggest that the cognitive aspects of interval generation (e.g., working memory, conscious time estimation) could still be taking place as learned during behavioral training even if it is no longer essential to receive reward.

### Conflict of interest statement

The authors declare that the research was conducted in the absence of any commercial or financial relationships that could be construed as a potential conflict of interest.
